# Reading Comprehension in the Arabic Diglossia: The SVR Between the Spoken and Literary Varieties

**DOI:** 10.1007/s10936-024-10054-z

**Published:** 2024-03-12

**Authors:** Ibrahim A. Asadi, Ronen Kasperski

**Affiliations:** 1 Department of Special Education and Learning Disabilities, The Academic Arab College for Education, 22 Hahashmal St., P.O. Box 8340, Haifa, Israel; 2 The Unit for the Study of Arabic Language, Edmond J. Safra Brain Research Center for the Study of Learning Disabilities, Faculty of Education, University of Haifa, Haifa, Israel; 3 Shaanan Academic Religious Teachers’ College, Haifa, Israel; 4 Gordon College of Education, Haifa, Israel

**Keywords:** Reading comprehension, Listening comprehension, Decoding, Arabic diglossia, Kindergarten

## Abstract

This study aimed to examine the validity of the “simple view of reading” (SVR) model in the diglossic Arabic language. Using a longitudinal design, we tested whether decoding and listening comprehension (LC) in kindergarten can later predict reading comprehension (RC) in the first grade and whether the contribution of LC to RC differs between the spoken and literary varieties of Arabic. The participants were 261 kindergartners who were followed to the first grade. Our results from separate SEM analysis for the spoken and literary varieties revealed some similarity between the explained variance in the spoken (52%) and literary (48%) variety models. However, while the contribution of LC to RC was higher than the contribution of decoding in the spoken variety model, an opposite pattern was observed in the literary variety model. The results are discussed in light of the diglossia phenomenon and its impact on comprehension skills in Arabic, with theoretical and pedagogical implications.

## Introduction

The “Simple View of Reading” (SVR), proposed by Gough and Tunmer (1986) and Hoover and Gough (1990), is one of the most prevalent models of reading comprehension (RC). This model posits that all variance in RC can be explained by decoding and oral language, usually tested by listening comprehension (Hogan et al., 2014). The validity of this model has been tested by different research studies in various languages (Asadi et al., 2017; Braze et al., 2015; Joshi et al., 2015); however, some have claimed that the SVR might be influenced by the unique characteristics of the language at hand (Florit & Cain, 2011).

The Arabic language is characterized, among other unique features, by a diglossic situation formed due to the existence of two forms of the same language—the spoken variety of Arabic that children use before beginning school and the literary (written) variety that children acquire formally when entering school—and therefore, different linguistic components of the literary language are not mature enough for reading acquisition (Saiegh-Haddad, 2003). Indeed, some researchers have argued that linguistic components of the literary version are less stored and represented in the mental lexicon (Saiegh-Haddad et al., 2011), which explains the low performance of Arabic-speaking children in reading skills in the PIRLS 2016 (Mullis et al., 2017). In the context of this study, the contribution of LC to RC in Arabic may differ from that of other languages where the oral and written languages do not differ from each other. Given the diglossic reality of Arabic-speaking children, this study aimed to challenge the SVR by examining the relative contribution of LC in kindergarten, separately tested in the spoken and literary varieties, to RC in the first grade.

RC is the ultimate goal of reading development and is considered a complex process that draws on different sources of knowledge (Perfetti & Stafura, 2014). Indeed, despite adequate instruction, readers at different ages still face difficulties in RC (Kendeou et al., 2016). Nevertheless, Gough and Tunmer (1986) claimed that the RC process is not so complicated; hence, they proposed the SVR model, which posits that RC is a product of decoding ability—the process of translating printed words into speech—and LC—the ability to understand oral language (Gough & Tunmer, 1986; Hoover & Gough, 1990). RC, moreover, necessitates the coordination of both a bottom-up level of decoding and word recognition and a top-down level of language comprehension and meaning processing.

The decoding skill is usually measured by pseudo-words and word recognition (Chen & Vellutino, 1997) that rely on grapho-phonemic conversion (GPC) processes in the early stages of learning to read. These processes are made possible by awareness to language sounds, i.e., phonological awareness that enables the readers to internalize the relations between the phonemes and the graphemes (i.e., orthographic symbols). However, this process is slow and insufficient for RC and must become automatic in order to allocate cognitive resources for higher functions of RC. Accordingly, for successful RC the reader needs to move from the slow process of grapho-phonemic conversion to a more efficient and advanced stage where larger orthographic units are identified. This transition depends on the orthographic knowledge and the orthographic patterns memorized by children. Several studies have provided evidence on the involvement of phonological awareness (Taibah & Haynes, 2010; Ziegler et al., 2010) and orthographic knowledge (Bekebrede et al., 2009; Elbeheri et al., 2011) in the basic skill of word reading.

LC represents the oral language construct that incorporates, along with different cognitive skills, all aspects of linguistic knowledge (Ouellette & Beers, 2009) needed for the processing and comprehension of presented information. Indeed, the importance of the LC component in the SVR model is based on the fact that skills that are needed for LC are basically the same as those needed for RC. The difference, however, is in how these skills are tested. While LC skills are tested and presented to the children orally, RC skills are tested and presented in the written modality (Hoover & Gough, 1990). Furthermore, some have claimed that LC as a general construct represents “all of verbal ability”, including the different linguistic components (Kirby & Savage, 2008). Indeed, the involvement of different linguistic components (e.g., syntax, vocabulary, morphology, and phonology) in LC was reported in different studies (Asadi, 2020, Farvardin & Valipouri, 2017; Fong & Ho, 2017; Fracasso et al., 2016; Kim, 2016).

The SVR model was explored in different languages by evaluating the contribution of decoding and LC to explaining the variance in RC and by comparing the relative contribution of each component. It was reported that the SVR explains between 25 and 83% of the variance in RC (Asadi et al., 2017; Florit & Cain, 2011; Primor et al., 2011). This wide range of explained variance in the SVR was related mainly to differences in languages and orthographic systems (Florit & Cain, 2011; Leppänen et al., 2008) and in the ages of the participants (Kershaw & Schatschneider, 2012). Taken together, previous findings suggest that decoding is more critical in the early stages of reading development and its contribution is thought to decrease with age as children become more skilled in their reading (Duke et al., 2004). An opposite pattern was reported concerning the contribution of LC, showing that its contribution tends to augment with age (Catts et al., 2005; Chen & Vellutino, 1997).

The relative contribution of decoding and LC to RC is influenced by the orthographic system of the language at hand. Specifically, given the consistency of the grapho-phonemic correspondence in transparent orthographies, children are believed to develop rapid phonological decoding skills, which in turn enable accurate reading by the end of first grade (Landi, 2010; Leppänen et al., 2008), decreasing the contribution of decoding. In deeper orthographies, however, the development of reading may be more difficult and slower than in transparent orthographies (Ziegler & Goswami, 2005); as a result, decoding skills may continue to play a critical role in RC for longer periods. Results supporting this view were reported in a recent study that compared the contribution of decoding and LC to RC in the transparent (vowelized) and deep (non-vowelized) Arabic orthographies in the 1st–2nd grade (Asadi & Ibrahim, 2018). Though the results from this transparent orthography showed that the contribution of decoding decreased with age, the contrary was observed in deep orthographies. Moreover, the contribution of LC to RC was higher in the transparent than in deep orthographies (Asadi & Ibrahim, 2018).

Although the validity of the SVR model was tested in relation to differences in the orthographic depth/transparency of the languages, which is related to the written language in this model, i.e., decoding, differences in LC were not the focus of the researchers. If LC represents the linguistic processes that form the basis of comprehension in oral language (Ouellette & Beers, 2009) and thus represents “all of verbal ability” (Kirby & Savage, 2008), the involvement of this component in RC may differ according to the proficiency of the learners’ oral language, which varies. This difference exists, for example, between monolinguals and other learners, such as those who learn a second language and minority learners (Mancilla-Martinez et al., 2011), where learners are thought to arrive at school with limited language skills needed for academic purposes. This may also be relevant for languages whose spoken variations use dialects from the written language, as in the case of diglossic Arabic.

The diglossia in Arabic refers to the existence of two varieties that are used in different situations (Ferguson, 1959): the spoken or literary varieties. The two forms of Arabic differ from each other in different linguistic aspects that include phonological, morphological, semantic, and syntactic knowledge (Asadi & Kawar, 2023; Saiegh-Haddad & Joshi, 2014), with linguistic structures affiliated with either the spoken or literary varieties (Saiegh-Haddad, 2007). Furthermore, while some researchers have considered the two forms of Arabic as two varieties of the same language, others have argued that they are two languages that are stored in separate lexicons (Ibrahim, 2008). For instance, different studies have shown that the phonological and lexical distance between the spoken and literary varieties of Arabic affects the performance of children in different phonological manipulations (Saiegh-Haddad, 2003, 2004), lexical access (Asaad & Eviatar, 2013; Asadi & Abu-Rabia, 2021), as well as in the comprehension of information in their oral language (Asadi et al., 2022). Consistently, the results of these studies showed an advantage in performance of the spoken language variety than of the literary one (Asadi & Ibrahim, 2018; Saiegh-Haddad, 2003, 2004). In this regard, the Lexical Quality Hypothesis (Perfetti, 2007) posits that different features and characteristics of words, including phonological, morphological, semantic, and syntactic, are representational properties of the mental lexicon. The quality of these representations is thought to be influenced by the experiences of the children in the oral language. Researchers have claimed that Arabic-speaking children enter school with a lack of readiness in various aspects of their literary oral language that is less represented in their mental lexicon (Saiegh-Haddad & Joshi, 2014).

In addition to the diglossic phenomenon, the Arabic orthographic system is considered a complex and unique orthography from different aspects (Taha, 2013). The Arabic orthography is an alphabetic system written from right to left, and it includes 29 letters of which three are long vowels. Three short vowels are added as diacritic marks above and under the letters. The Arabic orthography (similar to Hebrew) can be presented in both the transparent and deep versions due to the fact that transparency in the Arabic orthography is determined by the presence/absence of diacritic marks: the orthographic system is considered transparent when the short vowels are presented and provide the readers with the full phonological information and deep when the short vowels are absent (Abu-Rabia, 2001) and therefore many words become homographic (words that can be read correctly when pronounced in different ways). Children learn to read first with the vowelized/transparent orthography (with short vowels), while the unvowelized /deep version (without short vowels) is generally employed around the third-fourth grade (Asadi et al., 2017; Taha, 2013). Despite the fact that vowelized Arabic is considered a transparent orthography, there are several characteristics that influence the degree of orthographic transparency and contribute to some ambiguity in the relationship between letters and sounds. Of these, the presence of emphatic sounds that share the same phonology and rely on the same part of the articulation system, and the fact that 22 letters can be written in four different ways depending on their connectedness and place in the word, and finally the existence of sounds that in certain instances are written but not pronounced and the existence of letters that in some instances are pronounced but not written (Asadi et al., 2017).

Accordingly, in view of the unique characteristics of the Arabic language and particularly the challenging diglossia, the relative contribution of LC to RC may be reflected differently in Arabic than in other languages in which the oral language is consistent with the written language. Thus, the current study aims to shed light on differences related to LC that are thought to be influenced by the efficiency of the linguistic components of the oral language that differs between the spoken and literary varieties. The rationale behind testing decoding and especially LC in preschool was to control the diglossia effect by evaluating these components before children started their formal instruction in the literary language. Furthermore, using a longitudinal design and following the same children from kindergarten to the end of first grade, allowed us to examine the relative contribution of the two basic SVR components (decoding and LC skills) to RC in the first grade. Moreover, examining the prediction of these early literacy skills is important from an intervention perspective to allow early identification of children at risk for RC difficulties.

To test decoding in preschool, and following the previous study by Kendeou et al. (2013), we used decoding-related measures (hereafter decoding) that were found to be consistently strong predictors of decoding in different languages. LC was tested separately in kindergarten in the spoken and literary varieties to RC. Our main research question concerned the impact of diglossia in preschool on RC in the first grade. Specifically, we asked to what extent does the contribution of LC to RC differ between the spoken and literary varieties of Arabic. Given that the written language represents only the literary oral language, we predicted that LC in the literary variety would play a more important role in predicting RC than LC in the spoken variety. This prediction is based on the fact that today, parents and kindergarten teachers are more aware of the importance of enriching children's literary skills both in kindergarten and at home (Korat et al., 2013).

## Material and Methods

### Participants

The research participants consisted of 261 (126 girls) Arabic-speaking kindergartners. The participants were followed for one year, from the end of kindergarten (*M*_age in months_ = 71.4; *SD* = 3.7), i.e., before they received any formal reading instruction, to the end of first grade (*M*_age in months_ = 82.6; *SD* = 3.9). The participants were recruited from nine regular Arabic-speaking preschools in northern Israel, representing various socio-economic backgrounds. All children from the selected kindergartens participated in the study, aside from those reported by the school as having physical and mental disabilities. Written consent was obtained from the children’s parents. The research was approved by the ethical committee of the Arab Academic college in Haifa, Israel.

### Material

The tools used in this study included measures for assessing reading comprehension and listening comprehension (in the spoken and literary varieties). In addition, for the purpose of assessing the decoding skill in kindergarten, decoding-related measures, i.e., phonological awareness and orthographic processing, considered the most potent predictors of decoding in the early grades, were included (Asadi et al., 2017; Elbeheri et al., 2011; Taibah & Haynes, 2010). As for adaptation of the tasks to the participants’ age, 4–5 teachers from each grade level (in both kindergarten and first grade) checked and confirmed the suitability and the relevance of the content to the age of the participants.

#### Reading Comprehension

Three vowelized texts were devised for the reading comprehension task. The first was a narrative text on a *Stubborn Duke* and consisted of 41 words. The second was an informative text that related to *giraffes* and consisted of 64 words. The third was a narrative text that related to *Helping Each Other* and consisted of 68 words. Each text was followed by six multiple-choice questions that examined different levels of comprehension, including literal, interpretive, and applied levels. The text was available for the whole task in case children needed to re-read or to verify their responses and there were no time constraints. The participants received one point for each correct response, with a maximum score of 6 for each text. The reliability (Cronbach’s α) of the first and second texts was 0.78 and the reliability of the third text was 0.79.

#### Listening Comprehension

Two narrative texts were devised to test LC. The first text (Text A), *Play with Me*, contained 169 words. The second text (Text B), *The Sick Bear*, contained 267 words. Each text was presented to the children in both the spoken and literary varieties of Arabic. Specifically, for the purpose of controlling for the diglossic effect on LC, each text was tested twice in two different sessions: In the first session, Text A was presented in one version of Arabic (i.e., either spoken or literary), and the same text was tested in the second session (3–4 weeks later) in the other version. The same process was carried out for Text B. However, for the purpose of controlling for the effect of order, when tested with Text A half the participants started with the spoken version while the other half started with the literary version. The opposite order was followed when Text B was administered.

Fifteen multiple-choice questions were devised for each text in both versions. These questions tested different levels of comprehension and required both explicit and implicit processing. Immediately after the examiner read the text, the participants were required to answer multiple-choice questions that were read but also presented visually by the examiner. For each question, the participants were presented with four different pictures on which he/she was required to indicate the right response from among four options. The participants received one point for each correct response, with a maximum score of 15 for each text. The combined reliability (Cronbach’s α) for the two spoken LC texts was 0.81 and the combined reliability (Cronbach’s α) for the two literary texts was 0.83.

### Phonological Awareness Tests

Evaluation of phonological awareness was carried out using two lists that tested children’s performance in isolating initial and final phonemes orally (see Appendix 1 for a complete list of the stimuli).

#### Initial Phonemic Isolation

This task evaluates the children’s ability to isolate initial phonemes and consisted of a list of 32 items that were presented to the participants. The list comprised different types of items, including real words and pseudo-words. As for the length of the items, there were 8 simple monosyllabic items with three phonemes (e.g.,  which means 'dry'), 8 complex monosyllabic items with four phonemes (e.g.,  which means 'dust'), 8 bi-syllabic items with 5–6 phonemes (e.g.,  which means 'bright') and 8 disyllabic items with 7–8 phonemes (e.g.,  which means 'results'). The participant had to repeat each item after the examiner and to isolate the initial phonemes. For example, if the participant heard the word , after repeating the word he was required to pronounce the sound . One point was assigned for successfully isolating the target (isolated) phonemes, with a maximum score of 32. Responses with names instead of sounds were considered incorrect. Three training examples were given to ensure that children understood the instruction before beginning the test. The reliability of this test (Cronbach’s α) was 0.95.

#### Final Phonemic Isolation

The task consisted of a list of 32 items that was administered to children to test their ability to isolate final phonemes. This task comprised different types of items and lengths that were identical to those presented in the initial phonemic isolation task. The participant had to repeat each item after the examiner and to isolate the target phonemes. For example, if the participant heard the word , after repeating the word he was required to pronounce the sound . Similar to the previous task, one point was assigned for successfully isolating the final (isolated) phonemes, and thus the participant’s score was based on the total number of correctly answered items, with a maximum score of 32. Responses with names instead of sounds were considered incorrect. The reliability of this test (Cronbach’s α) was 0.96.

A general measure (index) of PA was produced from the two lists of the phonemic isolation task, with a maximum score of 64. This was done after calculating the correlation between the two tests (r = .64, *p* < .01). In addition, a confirmatory factor analysis (CFA) was carried out to assess the loadings of the two factors on the latent variable. The standardized loadings of the initial and final phoneme deletion tasks were.88 and.84, respectively.

### Orthographic Knowledge

Evaluation of orthographic knowledge was carried out using two subtests that examined letter identification and orthographic patterns.

#### Letter Identification

A list of 24 printed letters was used to evaluate knowledge of letters in Arabic. The participants were required to identify and mark the letter that was identical to the target from among four visual distractors (e.g., similar to the target letter but different in some features such as the orientation of the letters and the dots on/below the letters, their number, and their position). For example, the children were presented with the target letter of  on the right side of the row and were required to identify the identical letter from among the following: . The task was stopped after 3 min and the participant received one point for each correct response, with a maximum score of 24. The reliability of the test (α) was 0.83.

#### Orthographic Choice

This test examined the ability to identify wrong orthographic patterns (pseudo-orthography). A list of 32 items was used. Each item included three patterns that were presented to the children along a row. The participants were asked to identify and mark the incorrect form (“*the one that did not seem to be a real word*”) from among three distractors within 3 min. The incorrect forms included patterns with non-Arabic letters  and symbols that are not letters , as well as those with an illegal combination of letters and sequences . The task was stopped after 3 min and the participant’s score was based on the number of correctly marked incorrect forms, with a maximum score of 32. The reliability of the test (α) was 0.87.

A general measure (index) of orthographic knowledge was produced, with a maximum score of 56. This was done after calculating the correlation between the two tests (r = .54, *p* < .01). A confirmatory factor analysis (CFA) was carried out to assess the loadings of the two factors on the latent variable. The standardized loadings of the letter identification and orthographic choice tasks were .61 and .77, respectively.

### Procedure

The participants were individually examined in the presence of the examiner during school hours. Testing took place inside a quiet room at the preschools and schools that participated in this study. Given the ages of the participants, these tasks were administered in kindergarten in four sessions with an interval of about 3–4 weeks between sessions in order to test LC in the different versions (spoken vs. literary varieties). In the first grade, the three RC texts were tested in three different sessions. The different tasks were administered in different orders. All the examiners were students enrolled in a Master's degree program in linguistics and learning disabilities. For the purpose of the study and administration of the tasks, the examiners received detailed professional training. The study began in the third trimester of the school year, specifically, between April and June for kindergarten and in June for first grade. The examiners monitored completion of the research assignments and if there were children who did not complete assignments or were absent, the examiners scheduled another session to collect the missing data in order to avoid attrition and the handling of missing data.

## Results

Descriptive statistics of the observed variables of the participants’ scores in kindergarten and first grade are presented in Table 1. The means reflect the raw scores for achievements in all variables. Performance was acceptable in all variables and there were no indications of floor or ceiling effects. A correlation analysis was conducted between all observed variables. As presented in Table 2, the correlation analysis showed significant correlations (*p* < .01) between all variables, with no indication of multi-co-linearity effects. Specifically, the highest correlations were observed between the different RC scores and to some extent between the different LC scores. The correlation between RC and LC in the spoken variety was slightly higher than in the literary variety.

**Table 1:** Descriptive statistics of raw scores mean and SD

Variables	*M*	*SD*	*Max*
*Kindergarten*			
Initial phonemic isolation	20.6	9.2	32
Final phonemic isolation	19.9	10.5	32
PA^a^	41.1	11.3	64
Letter similarity	19.9	3.8	24
Orthographic choice	21.8	5.9	32
Orthographic knowledge^b^	41.7	8.4	56
LC spoken text A	10.5	3.0	15
LC spoken text B	9.9	3.1	15
LC literary text A	9.4	3.1	15
LC literary text B	8.7	2.9	15
*First grade*			
RC text A	3.9	1.6	6
RC text B	3.5	1.6	6
RC text C	3.1	1.7	6

*LC* listening comprehension, *RC* reading comprehension

^a^PA total (i.e., sum index of initial phonemic isolation and final phonemic isolation)

^b^Orthographic knowledge (i.e., sum index of letter similarity and orthographic choice)

**Table 2:** Correlation analyses for observed variables in kindergarten and first grade

Variables	1	2	3	4	5	6	7	8	9
*Kindergarten*									
1. Phonological awareness	–								
2. Orthographic knowledge	.51	–							
3. LC spoken A	.35	.38	–						
4. LC spoken B	.37	.37	.53	–					
5. LC literary A	.34	.33	.56	.38	–				
6. LC literary B	.29	.32	.50	.64	.52	–			
*First grade*									
7. RC text A	.40	.32	.44	.37	.28	.34	–		
8. RC text B	.42	.30	.41	.34	.33	.35	.57	–	
9. RC text C	.40	.30	.34	.35	.32	.32	.61	.58	–

*LC* listening comprehension, *RC* reading comprehension

In order to assess the relative contribution of decoding-related measures and LC to RC, a Structural Equation Modeling (SEM) analysis was conducted. Maximum likelihood estimation procedures were used to analyze the variance of the predictors (latent variables) using AMOS 20.0 (Arbuckle, 2011), separately for the spoken and literary versions of LC. The goodness of fit indices used were the Root Mean Square Error of Approximation (RMSEA), the Comparative Fit Index (CFI), and the Tucker-Lewis Index (TLI). As indicated by the fit indices, the SEM model provided a good fit with the data for the spoken variety (RMSEA = .03, CFI = .99, TLI = .98) and for the literary one (RMSEA = .01, CFI = .99, TLI = .99)..

**Fig. 1:**
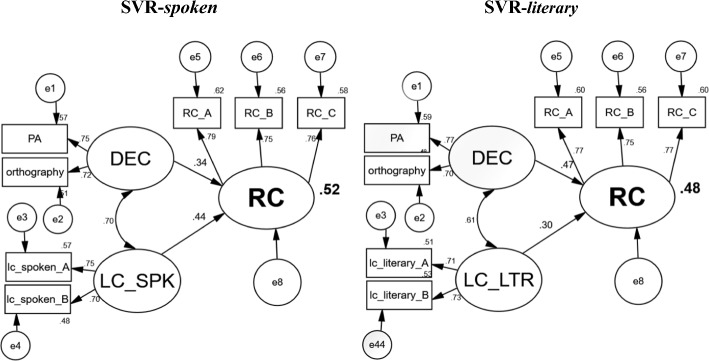
.

Two SEM models were carried out separately for the spoken and literary varieties. The results of this analysis showed that the latent variables of both decoding (represented by phonological awareness and orthographic knowledge) and LC in kindergarten explained 52% of the variance in RC in the first grade in the spoken variety and 48% in the literary variety (see Fig. 1). The contributions of these latent independent variables to RC were significant in the two models. However, despite the high similarity between the percentages of variance in the spoken and literary variety models, the contribution of the independent variables, shown by the standardized coefficients (*Beta-β*), indicated opposite patterns between the spoken and literary models. Using the Fisher *z* transformation (Meng et al., 1992) for comparing the correlated coefficients, we analyzed the relative contribution of each predictor (Decoding vs. LC) to accounting for the dependent variable (RC) in each model. In the SVR-spoken variety model, the contribution of LC to RC was significantly higher (*β* = .44) than that of decoding to RC (*β* = .34; z = 2.23, *p* < .05). In contrast, in the SVR-literary variety model, the contribution of decoding to RC was significantly higher (*β* = .47) than that of LC to RC (*β* = .30; z = 3.34, *p* < .001). These results suggest that LC is more dominant than decoding in spoken Arabic, whereas decoding is more dominant than LC in literary Arabic.

## Discussion

The primary goal of this study was to test the validity of the SVR in the diglossic Arabic language and to analyze whether or not the involvement of decoding-related measures and LC in RC differs between the spoken and literary Arabic varieties. Our findings from the SEM analysis, using a longitudinal design, provided support for the SVR model, which showed that both decoding-related measures and LC in kindergarten explained a moderate portion of the variance in RC in the first grade in both models of the spoken and the literary varieties (52% and 48%, respectively). However, the relative contribution of the two basic components of this model was reflected differently in the spoken and literary models.

Regardless of the differences between the spoken and literary varieties of Arabic, the support they provided to the SVR model in this study is compatible with previous studies of different languages in the first grade (Asadi & Ibrahim, 2018; Ouellette & Beers, 2009) and in other grades as well (Braze et al., 2015; Joshi & Aaron, 2000; Kershaw & Schatschneider, 2012; Primor et al., 2011), showing that both decoding-related measures and LC are significant contributors to RC, with more modest explained variance than in English and other languages. Unlike previous studies, especially in the Arabic language, in this study the validity of the SVR and the contribution of decoding-related measures and LC was tested and proven already in kindergarten, i.e., before children started their formal reading instruction, showing that both components play a critical role in kindergarten as emergent contributors to RC in the first grade. While some authors have emphasized that the contribution of LC is more significant as students become more skilled readers, our results show that LC is critical even before reading acquisition. This finding might be explained by the fact that there are general shared comprehension processes for both RC and LC that rely on the same linguistic and cognitive skills (Diakidoy et al., 2005). Another explanation may be related to the complexity of the Arabic orthographic system (Ibrahim et al., 2002) that seems to obligate the reader, even in the early stages of reading development, to rely on the oral language (represented by listening comprehension), more than on the decoding component of the SVR, for successful RC.

As for the impact of the Arabic diglossia on RC and on the SVR model, the results obtained from the SEM models showed some similarity in the explained variance in RC between the spoken and literary models, with a slight advantage for the spoken model (52%) than for the literary one (48%). This disadvantage of the literary language in explaining variance in RC over the spoken one was contrary to our prediction. This unexpected finding might be related to the fact that, unlike the spoken language, linguistic components of the literary language are poorly represented in the mental lexicon during this early stage of development (Saiegh-Haddad et al., 2011). The poor representation of the literary variety in the mental lexicon is considered a further obstacle to the efficient execution of the linguistic components and processes necessary for RC. As a result, readers are required to allocate more cognitive resources at the cost of higher-order processes of RC. Additionally, the similarity in the explained variance between spoken and literary language may support the view that although the spoken and literary varieties differ in various linguistic aspects, they appear to be more strongly linked to each other (Asadi & Kawar, 2023) with a common rather than separate lexicons (Ibrahim, 2008).

Despite the similarity in the explained variance of the two models, the contribution of the basic components of the spoken and literary models to RC differed. While the contribution of LC was stronger than decoding in the spoken model, this contribution decreased in the literary model, where decoding became the most dominant contributor to RC. These results may be explained by the lexical quality hypothesis (Perfetti, 2007) and by the availability of linguistic components of the oral language and their representation in the mental lexicon. Given that preschoolers mainly use the spoken variety for their daily oral communication purposes, the linguistic components of the spoken language are thought to be much better represented in the mental lexicon than those of the literary language. Therefore, processing oral language that is available to the children is thought to be more efficient for understanding and building a successful mental model of the text, which was reflected in this study by the dominant contribution of LC to RC in the spoken model. When these linguistic components are less represented, however, as in the literary variety model, the contributions of oral language decrease in favor of decoding.

In summary, this study provides support for the SVR model in the diglossic Arabic language, showing that decoding-related measures and LC (whether assessed in spoken or literary varieties) in preschool predict RC a year later. Further, the present study highlights the role of differences related to oral language rather than to other factors such as the depth/transparency of the orthographic systems, as suggested in other studies, bear theoretical implications. Testing the validity of the SVR model in different languages should thus take into consideration, in addition to the differences in the orthographic systems, the differences in the oral language and its representation in the mental lexicon. Also, a considerable portion of variance was not explained, suggesting that the unique characteristics of languages and additional variables aside from decoding and LC should be considered in further studies, especially in the preschool stages. Theoretically, characterizing emergent linguistic and cognitive variables on the basis of future complex and important comprehension skills may enhance the very early identification of children who are at risk for reading comprehension and, most importantly, before children face problems in RC at school. This may prevent future frustration and failure experiences of children in their reading acquisition. As for the pedagogical implications, the findings of our study illustrate how important it is that children experience the literary language in early stages of preschool. Early exposure to the literary variety should improve the quantity and quality of the representation of the literary linguistic component in the mental lexicon and contribute to more successful access to and retrieval of these representations. As a result, cognitive resources are retained for the benefit of higher order processes such as RC. Finally, given the importance of the spoken oral language variety for RC and despite the need to enrich the literary language variety in preschool, the spoken language should not be ignored and teachers must continue working with the spoken language as well, especially since the two varieties of Arabic seem to enrich each other.

This study has several limitations. We would like to emphasize that in light of the diglossic nature of the Arabic language, our intention was to validate the original SVR model proposed by Gough and Tunmer (1986) and Hoover and Gough (1990) in both the spoken and literary Arabic varieties. Originally, the SVR model posits that all the variance in RC can be explained by decoding and oral language, usually tested by listening comprehension (Hogan et al., 2014). Although other researchers (i.e., Joshi et al., 2015) elaborated the basic SVR model and included other linguistic variables beyond the two basic components, this was not within the scope of the current research. Thus, future studies should elaborate the basic model from the current study and add expressive and receptive measures of syntax, vocabulary, and listening in their latent variables. Furthermore, additional decoding-related measures should be considered in future studies.

## Appendix

In each category, the first two items are in the literary Arabic variety, the third and fourth items are identical in the spoken and literary varieties, the fifth and sixth items are in the spoken variety, and the seventh and eighth items are pseudo-words.
